# Combined High Resistin and EGFR Expression Predicts a Poor Prognosis in Breast Cancer

**DOI:** 10.1155/2020/8835398

**Published:** 2020-11-28

**Authors:** Yue Zeng, Chih-Hsin Tang, Yan Wang, Hua-Jun Lu, Bi-Fei Huang, Qian Wang, Jun-Kang Shao, Lu-Lu Jin, Chao-Qun Wang, Li-Song Teng

**Affiliations:** ^1^Department of Surgical Oncology, The First Affiliated Hospital, Medical School of Zhejiang University, Hangzhou, Zhejiang, China; ^2^Department of Pathology, Affiliated Dongyang Hospital of Wenzhou Medical University, Dongyang, Zhejiang, China; ^3^Graduate Institute of Basic Medical Science, China Medical University, Taichung, Taiwan; ^4^Department of Pharmacology, School of Medicine, China Medical University, Taichung, Taiwan; ^5^Department of Biotechnology, College of Health Science, Asia University, Taichung, Taiwan; ^6^Department of Medical Oncology, Affiliated Dongyang Hospital of Wenzhou Medical University, Dongyang, Zhejiang, China; ^7^Department of Oncological Radiotherapy, Affiliated Dongyang Hospital of Wenzhou Medical University, Dongyang, Zhejiang, China; ^8^Laboratory of Biomedicine, Affiliated Dongyang Hospital of Wenzhou Medical University, Dongyang, Zhejiang, China

## Abstract

Elevated levels of resistin and epidermal growth factor receptor (EGFR) facilitate the development of breast cancer, although there are no reports of any correlation between these proteins. This study analyzed 392 human breast cancer tissue specimens and 42 samples of adjacent normal tissue. Rates of positive and strongly positive resistin expression were significantly higher in breast cancer tissue than in the adjacent nontumor tissue (83.2% vs. 23.8% and 20.9% vs. 0.0%, respectively; *P* < 0.001 for both comparisons). Positive resistin expression was significantly associated with tumor size, grade, stage, estrogen receptor (ER), progesterone receptor (PR), human epidermal growth factor receptor 2 (HER2) status, and molecular classification; strongly positive resistin expression was associated with tumor grade, ER, PR, HER2 status, and molecular classification. Significantly positive correlations were observed between positive and strongly positive resistin expression and corresponding levels of EGFR expression. Relapse-free and overall survival was worse for patients with high levels of both proteins than for those with high levels of only one protein or normal levels of both proteins. Our evidence suggests that combined high levels of resistin and EGFR expression correlate with survival in patients with breast cancer.

## 1. Introduction

Elevated levels of circulating resistin have been linked to a higher risk of breast cancer [1, 2], and significantly elevated serum resistin has been documented in patients with breast cancer [3–6]. Significant correlations have been observed between high resistin expression in breast cancer tissue and tumor stage, tumor size, lymph node metastasis, estrogen receptor (ER) status, and poor survival [7]. We have previously reported finding much higher levels of resistin expression in breast cancer tissue than in normal breast tissue [8], but in that study, we did not analyze the relationship between the high expression of resistin and the clinicopathological characteristics of breast cancer patients.

Epidermal growth factor receptor (EGFR), a member of the HER family of receptor tyrosine kinases, is abnormally expressed and activated in many epithelial tumors and plays a critical role in the initiation and development of cancer via modulating downstream signaling pathways [9, 10]. Our previous investigations echo other reports describing how resistin and EGFR promote tumor progression through downstream p44/p42 MAPK (ERK1/2) signaling [11, 12]. Resistin can also increase EGFR phosphorylation through the Toll-like receptor 4 (TLR4)/Src pathway and thereby promote lung adenocarcinoma metastasis [13]. However, no reports exist as to any association between resistin and EGFR in breast cancer.

This paper describes our insights derived from immunohistochemistry (IHC) analyses of resistin and EGFR expression in breast cancer and healthy normal breast tissue specimens obtained from 392 Chinese Han women. We detail the clinical prognostic significance of a positive association between resistin and EGFR expression in breast cancer.

## 2. Materials and Methods

### 2.1. Patients and Tissue Samples

Breast cancer tissue samples were obtained from 392 untreated Chinese Han women aged 24–90 years (median 50 years) who underwent surgery in the Affiliated Dongyang Hospital of Wenzhou Medical University (Dongyang, Zhejiang, China) between 2007and 2019. Forty-two samples of adjacent normal breast tissue were also obtained following surgical resection. Pathohistological diagnoses followed the World Health Organization breast tumor classification criteria [14]. Histological grading was based on the Scarff-Bloom-Richardson system [15]. According to ER, progesterone receptor (PR), human epidermal growth factor receptor 2 (HER2), and Ki-67 status, the samples were classified into 4 molecular subtypes [11, 16, 17]: luminal A (ER^+^/PR^+^[≥20%]/HER2^–^, Ki‐67 < 14%); luminal B, containing hormone receptor-positive cases that did not meet the conditions of luminal A; HER2-enriched (ER^–^, PR^–^, and HER2^+^); or triple-negative breast cancer (TNBC) (ER^–^, PR^–^, and HER2^–^). Follow-up information was available for 239 breast cancer patients with a median follow-up time of 60 months (range, 6–72 months). The Ethics Committee of the Affiliated Dongyang Hospital of Wenzhou Medical University approved this study. All study methods satisfied the relevant guidelines and regulations issued by the Affiliated Dongyang Hospital of Wenzhou Medical University.

### 2.2. Tissue Array Preparation

We followed the methods described by Wang et al. [18].

### 2.3. IHC Analysis

We followed the methods of Wang et al. [18]. The primary antibodies consisted of anti-resistin mouse monoclonal antibody (clone C-10, diluted at 1 : 25; Santa Cruz Biotechnology, Santa Cruz, USA), anti-EGFR rabbit polyclonal antibody (clone 1005, diluted at 1 : 100; Santa Cruz Biotechnology), anti-ERK1/2 rabbit monoclonal antibody (clone EPR18444, diluted at 1 : 1000; Abcam, Cambridge, England), ready-to-use anti-ER rabbit monoclonal antibody (clone SP1, Dako), ready-to-use anti-PR mouse monoclonal antibody (clone PgR636, Dako), HercepTest (Dako), and ready-to-use anti-Ki-67 mouse monoclonal antibody (clone MIB-1, Dako).

### 2.4. Assessment of Staining

The entire section was scanned and scored independently by 2 pathologists. Resistin and EGFR expression staining intensity was scored on a 4-point scale from 0 (negative) to 1 (weak), 2 (moderate), or 3 (strong) [18]. A case was recorded as resistin-positive if the cytoplasmic staining intensity of positive invasive cancer cells was 2 or 3; 3 was deemed strongly positive [8]. Cases were EGFR-positive when ≥10% of the invasive tumor cells had a membranous staining intensity of 1, 2, or 3; 2 and 3 were considered to be strongly positive [11]. ER or PR positivity was satisfied if the percentage of positive invasive cancer cells (nuclear staining) was ≥1% [19]. HER2 status was determined by the 2018 American Society of Clinical Oncology/College of American Pathologists guidelines for HER2 testing in breast cancer [20]. High levels of resistin and EGFR expression were expressed as positive or strongly positive for resistin and strongly positive for EGFR.

### 2.5. Patient Follow-Up

We followed the methods of Wang et al. [18].

### 2.6. Statistical Analysis

We followed the methods of Wang et al. [18]. Multivariate analysis using the Cox proportional hazard model was performed to investigate independent factors prognostic of RFS and OS.

## 3. Results

### 3.1. Expression of Resistin in Breast Tissue and Its Relationship with Clinicopathological Variables

The rate of positive resistin expression in breast cancer tissue specimens was 83.2% (326/392), which included 82 (20.9%) strongly positive cases; corresponding rates in normal breast tissue specimens were 23.8% (10/42) and 0.0% (0/42), respectively. Rates of positive and strongly positive resistin expression were significantly higher in breast cancer tissue than in normal breast tissue (*P* < 0.001 and *P* = 0.001, respectively) (Table 1). As shown in Table 2, we observed significant associations in breast cancer tissue specimens between positive resistin expression and several clinical parameters, including tumor size (*P* = 0.012), grade (*P* < 0.001), stage (*P* = 0.042), ER (*P* < 0.001) and PR (*P* < 0.001) status, HER2 status (*P* = 0.021), and molecular classification (*P* < 0.001). Strongly positive resistin expression was associated with higher tumor grade (*P* < 0.001), ER (*P* < 0.001) and PR (*P* < 0.001) status, HER2 status (*P* = 0.001), and molecular classification (*P* < 0.001).

### 3.2. Correlation of Resistin and EGFR Expression in Breast Cancer

**Figure 1:**
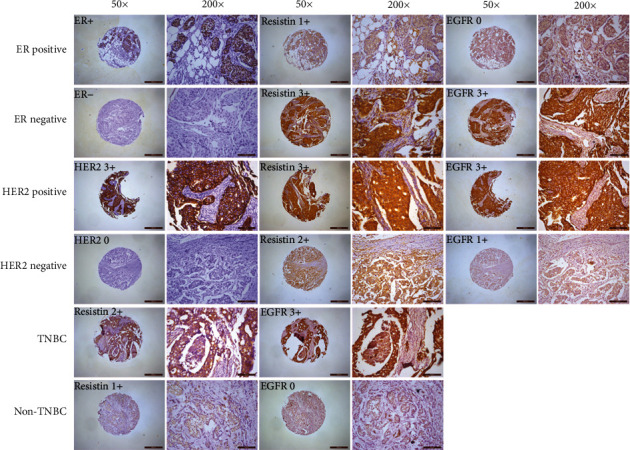
Resistin and EGFR protein expression was analyzed in ER-positive/ER-negative, HER-positive/HER-negative, TNBC/non-TNBC human breast cancer tissues by immunohistochemical staining. Cancer tissue microarrays were immune-stained with anti-resistin, anti-EGFR, anti-ER, and anti-HER2 antibodies. Representative images of stained tissues are shown.

**Figure 2:**
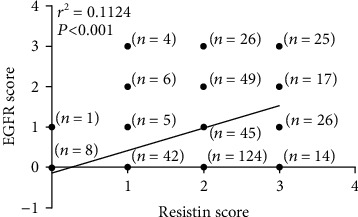
A positive correlation was observed between levels of resistin and EGFR expression in breast cancer tissues.

Rates of positive and strongly positive EGFR expression in breast cancer tissues were 52.0% (204/392) and 32.4% (127/392), respectively. When we analyzed the relationship between resistin and EGFR expression in breast cancer tissue specimens, we found significantly higher levels of positive or strongly positive EGFR expression among resistin-positive cases (57.7% [188/326] and 35.9% [117/326], respectively) compared with resistin-negative cases (24.2% [16/66] (*P* < 0.001) and 15.2% [10/66] (*P* = 0.001), respectively, Table 3). Spearman correlation analysis revealed significantly positive correlations between positive levels of resistin expression and EGFR-positive or strongly positive expression in breast cancer tissue specimens (*r* = 0.250 and *P* < 0.001 and *r* = 0.166 and *P* = 0.001, respectively). Similarly, as shown in Table 4 and Figure 1, significantly higher levels of positive or strongly positive EGFR expression were identified in cases that were strongly positive for resistin (82.9% [68/82] and 51.2% [42/82], respectively) compared with those that were not strongly resistin-positive (43.9% [136/310] (*P* < 0.001) and 27.4% [85/310] (*P* < 0.001), respectively). Spearman correlation analysis revealed significantly positive correlations between strongly positive levels of resistin expression and EGFR-positive or strongly positive expression in breast cancer tissue specimens (*r* = 0.318 and *P* < 0.001 and *r* = 0.207 and *P* < 0.001, respectively). Similarly, a positive correlation was observed between staining intensity scores of resistin and EGFR in breast cancer tissues (*r*2 = 0.1124, *P* < 0.001) (Figure 2).

### 3.3. Combined High Resistin and EGFR Expression Is Associated with Worse Survival in Breast Cancer

**Figure 3:**
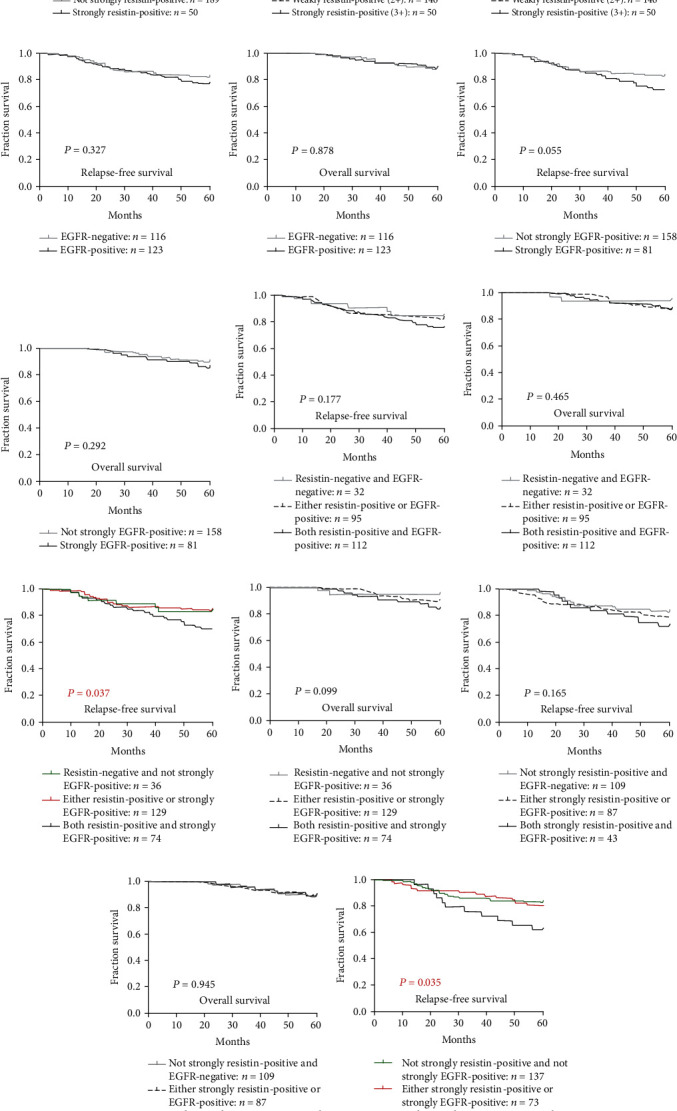
Levels of resistin and EGFR expression were associated with survival of patients with breast cancer. Associations were analyzed between resistin-positive expression and (a) relapse-free survival (RFS) and also (b) overall survival (OS). Associations were analyzed between strongly positive resistin expression and (c) RFS and also (d) OS. Associations were analyzed between strongly positive and weakly-positive resistin expression and (e) RFS and also (f) OS. Associations were analyzed between EGFR-positive expression and (g) RFS and also (h) OS. Associations were analyzed between strongly positive EGFR expression and (i) RFS and also (j) OS. Kaplan-Meier curves for (k) RFS and (l) OS in breast cancer patients with combined resistin-positive and EGFR-positive expression. Kaplan-Meier curves for (m) RFS and (n) OS in breast cancer patients with combined resistin-positive and strongly positive EGFR expression. Kaplan-Meier curves for (o) RFS and (p) OS in breast cancer patients with combined strongly positive resistin and EGFR-positive expression. Kaplan-Meier curves for (q) RFS and (r) OS in breast cancer patients with combined strongly positive resistin and strongly positive EGFR expression. *P* values were calculated using the Mantel-Cox log-rank test.

RFS and OS were worse in patients with resistin-positive tumors compared with those who were resistin-negative, and RFS was worse in patients with strongly resistin-positive tumors compared with those whose tumors were not strongly resistin-positive, while patients whose tumors were strongly resistin-positive experienced worse RFS compared with patients whose tumors were weakly resistin-positive and those with resistin-negative tumors (*P* = 0.237, *P* = 0.128, *P* = 0.171, and *P* = 0.105, respectively) (Figures 3(a)–3(f)). Similarly, RFS was worse for EGFR-positive patients compared with EGFR-negative patients, while RFS and OS were worse for strongly EGFR-positive patients compared with those without strongly EGFR-positive tumors (*P* = 0.327, *P* = 0.055, and *P* = 0.292, respectively) (Figures 3(g)–3(j)).

As shown in Figures 3(k)–3(n), patients whose primary tumors were both resistin-positive and strongly EGFR-positive (*n* = 74) had a mean RFS of 51.5 months (an estimated 5-year RFS rate of 68.9%); patients whose tumors were either resistin-positive or strongly EGFR-positive (*n* = 129) had a mean RFS of 54.3 months (an estimated 5-year RFS rate of 83.7%), while patients whose tumors were resistin-negative and not strongly EGFR-positive (*n* = 36) had a mean RFS of 54.1 months (an estimated 5-year RFS rate of 83.3%, *P* = 0.037). Patients whose tumors were both resistin-positive and strongly EGFR-positive experienced worse OS compared with patients whose tumors were either resistin-positive or strongly EGFR-positive and patients whose tumors were resistin-negative and not strongly EGFR-positive (*P* = 0.099).

As shown in Figures 3(o)–3(r), patients whose primary tumors were both strongly resistin-positive and strongly EGFR-positive (*n* = 29) had a mean RFS of 49.1 months (an estimated 5-year RFS rate of 62.1%); patients whose tumors were either strongly resistin-positive or strongly EGFR-positive (*n* = 73) had a mean RFS of 54.2 months (an estimated 5-year RFS rate of 79.5%), and patients whose tumors were not strongly resistin-positive and not strongly EGFR-positive (*n* = 137) had a mean RFS of 53.9 months (an estimated 5-year RFS rate of 82.5%, *P* = 0.035). Patients whose tumors were both strongly resistin-positive and strongly EGFR-positive experienced worse OS compared with patients whose tumors were either strongly resistin-positive or strongly EGFR-positive and patients whose tumors were not strongly positive for either protein (*P* = 0.453). Cox proportional hazards regression analysis did not reveal any significant associations between resistin-positive and strongly EGFR-positive tumor tissue (hazard ratio (HR) = 0.736, 95%CI = 0.198–2.734, *P* = 0.648; 0.765, 0.110–5.323, *P* = 0.786), strongly resistin-positive and strongly EGFR-positive tumor tissue (0.784, 0.273–2.255, *P* = 0.652; 0.359, 0.100–1.294, *P* = 0.118), and RFS or OS.

### 3.4. Combined High Resistin and EGFR Expression Is Associated with Survival in Non-TNBC or TNBC, ER-Negative or ER-Positive, HER-Negative or HER2-Positive Breast Cancer

**Figure 4:**
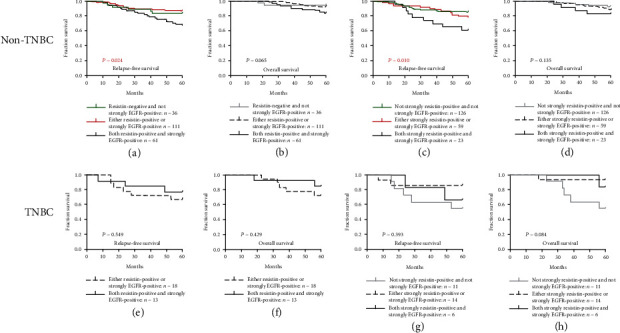
Combined high levels of resistin and EGFR are associated with survival in non-TNBC and TNBC disease. Kaplan-Meier curves for (a) RFS and (b) OS in non-TNBC patients with combined resistin-positive and strongly positive EGFR expression. Kaplan-Meier curves for (c) RFS and (d) OS in non-TNBC patients with combined strongly positive resistin and strongly positive EGFR expression. Kaplan-Meier curves for (e) RFS and (f) OS in TNBC patients with combined resistin-positive and strongly positive EGFR expression. Kaplan-Meier curves for (g) RFS and (h) OS in TNBC patients with combined strongly positive resistin and strongly positive EGFR expression. *P* values were calculated using the Mantel-Cox log-rank test.

We analyzed the effect of combined high expression of resistin and EGFR on the prognosis of non-TNBC or TNBC, ER-negative or ER-positive, HER-negative or HER2-positive tumors. As shown in Figures 4(a) and 4(b), non-TNBC patients whose primary tumors were both resistin-positive and strongly EGFR-positive (*n* = 61) had a mean RFS of 51.3 months (an estimated 5-year RFS rate of 67.2%); patients whose tumors were either resistin-positive or strongly EGFR-positive (*n* = 111) had a mean RFS of 55.2 months (an estimated 5-year RFS rate of 86.5%), while patients whose tumors were resistin-negative and not strongly EGFR-positive (*n* = 36) had a mean RFS of 54.1 months (an estimated 5-year RFS rate of 83.3%, *P* = 0.024). Non-TNBC patients whose tumors were both resistin-positive and strongly EGFR-positive experienced worse OS compared with patients whose tumors were either resistin-positive or strongly EGFR-positive and also patients whose tumors were resistin-negative and not strongly EGFR-positive (*P* = 0.065). As shown in Figures 4(c) and 4(d), non-TNBC patients whose primary tumors were both strongly resistin-positive and strongly EGFR-positive (*n* = 23) had a mean RFS of 48.3 months (an estimated 5-year RFS rate of 60.9%); patients whose tumors were either strongly resistin-positive or strongly EGFR-positive (*n* = 59) had a mean RFS of 54.5 months (an estimated 5-year RFS rate of 78.0%), and patients whose tumors were not strongly resistin-positive and not strongly EGFR-positive (*n* = 126) had a mean RFS of 54.6 months (an estimated 5-year RFS rate of 84.9%, *P* = 0.010). Non-TNBC patients whose tumors were both strongly resistin-positive and strongly EGFR-positive experienced worse OS compared with patients whose tumors were either strongly resistin-positive or strongly EGFR-positive and patients whose tumors were not strongly positive for either protein (*P* = 0.135).

As shown in Figures 4(e)–4(h), in TNBC, the prognosis of tumors that were both resistin-positive and strongly EGFR-positive, or strongly resistin-positive and strongly EGFR-positive, did not differ significantly from that of other breast cancer groups. Similarly, in ER-negative or ER-positive and HER2-negative or HER2-positive disease, the prognosis of tumors that were both resistin-positive and strongly EGFR-positive, or strongly resistin-positive and strongly EGFR-positive, did not differ significantly from that of other groups.

## 4. Discussion

The cytokine resistin participates in several physiological and pathological processes, including metabolism, inflammation, autoimmunity, and various cancers, including breast cancer [1–6, 21–24]. Higher levels of circulating resistin have been linked to a risk of developing breast cancer [1, 2], and significantly elevated resistin expression has been documented in patients with breast cancer [3–7]. Our previous study found upregulated resistin expression in breast cancer tissue than in normal breast tissue [8], but in that study, we did not analyze the relationship between the high expression of resistin and the clinicopathological characteristics of breast cancer patients. In this study, we observed that both positive and strongly positive rates of resistin expression are significantly higher in breast cancer tissue specimens compared with normal breast tissue and that both positive and strongly positive resistin expressions are significantly associated with a number of clinicopathological parameters in breast cancer patients. For example, positive resistin expression was correlated with larger tumor size, higher tumor grade, higher clinical stage, and HER2-positive expression; strongly positive resistin expression was correlated with higher tumor grade and HER2-positive expression. Since higher tumor grade and HER2-positive expression are known to be more aggressive and to have a poor prognosis [15, 25], this finding indicates that the high expression of resistin may be closely related to highly invasive breast cancer and a poor prognosis. Our results also revealed significantly increased levels of resistin expression in ER- and PR-negative disease, and evidence has shown that resistin promotes invasion and migration of the TNBC cell line MDA-MB-231 [26]. Since hormone receptor-negative breast cancer patients are unsuitable candidates for endocrine therapy, further research should examine whether these patients may benefit from the downregulation of resistin. We also found in this study that resistin is highly expressed in TNBC and HER2-enriched subtypes. TNBC is associated with a more aggressive clinical course and poorer prognosis than other types of breast cancer [27–30]. Our findings suggest that resistin may be a potential marker for the treatment of patients with TNBC.

Our previous study have shown that EGFR promotes breast cancer invasion through downstream p44/p42 MAPK (ERK1/2) signaling [11], and resistin is known to enhance angiogenesis in human osteosarcoma cells via the ERK1/2 signaling [12]. In this study, our data revealed a significantly positive correlation between positive levels of resistin and ERK1/2 in breast cancer tissues (Supplementary Materials, Tables S1 and S2). Another study has shown that resistin activates Toll-like receptor 4 (TLR4) and engages with the Src pathway which activated the EGFR phosphorylation and increased epithelial-mesenchymal transition (EMT), subsequently inducing the migration and invasion of lung adenocarcinoma [13]. In addition, resistin facilitates breast cancer progression via TLR4/nuclear factor kappa-light-chain-enhancer of activated B cells (NF-*κ*B)/signal transducer and activator of transcription 3 (STAT3) signaling pathway-mediated induction of mesenchymal phenotypes and stemness properties [31]. These results suggest that both resistin and EGFR high expression tumor has more potent tumor metastasis and recurrence, and there may be a relationship between resistin and EGFR expression in breast cancer. In this study, we found that tumors that are resistin-positive or strongly resistin-positive are significantly positively correlated with EGFR-positive and strongly EGFR-positive expression. We therefore sought to determine the clinical prognostic significance of our observed highly significant correlation between resistin and EGFR expression in breast cancer tissue.

In this study, both resistin and EGFR impacted adversely upon survival in breast cancer, but neither protein alone had a significant impact. Breast cancer patients whose tumors were both resistin-positive and strongly EGFR-positive, or were both strongly resistin-positive and strongly EGFR-positive, had worse RFS than all other breast cancer patients. In the non-TNBC and TNBC subgroup analyses, similar results were observed with non-TNBC, but not with TNBC. This may be due to the fact that there were too few cases for analysis. Further study should examine the effects of combined high resistin and EGFR expression on the prognosis of patients with TNBC. These results suggest that resistin and EGFR are potential clinical biomarkers of disease progression and prognosis in breast cancer and that simultaneously targeting these proteins is a potentially useful therapeutic strategy in this disease.

## Figures and Tables

**Table 1:** Resistin expression in breast tissue specimens obtained from 392 Chinese Han patients.

Tissue	No.	Resistin expression			
		Negative *n* (%)	Positive *n* (%)	Not strongly positive *n* (%)	Strongly positive *n* (%)
Normal	42	32 (76.2%)	10 (23.8%)	42 (100.0%)	0 (0.0%)
Tumor	392	66 (16.8%)	326 (83.2%)^∗^	310 (79.1%)	82 (20.9%)^∗∗^

^∗^ *P* < 0.001; ^∗∗^*P* = 0.001.

**Table 2:** Association of resistin expression with clinicopathological parameters in 392 Chinese Han patients with breast cancer.

Parameters	No. of patients	Positive resistin expression *n* (%)	*P* value	Strongly positive resistin expression *n* (%)	*P* value
*Age (years)*					
≤35	19	16 (84.2%)	0.966	6 (31.6%)	0.466
35–55	238	197 (82.8%)		47 (19.7%)	
>55	135	113 (83.7%)		29 (21.5%)	
*Tumor size (cm)*					
≤2	181	140 (77.3%)	0.012	33 (18.2%)	0.466
2–5	192	168 (87.5%)		45 (23.4%)	
>5	19	18 (94.7%)		4 (21.1%)	
*Lymph node metastases*					
No	199	159 (79.9%)	0.080	45 (22.6%)	0.402
Yes	193	167 (86.5%)		37 (19.2%)	
*Tumor grade*					
I	19	11 (57.9%)	<0.001	0 (0.0%)	<0.001
II	265	215 (81.1%)		47 (17.7%)	
III	108	100 (92.6%)		35 (32.4%)	
*Tumor stage*					
I	106	80 (75.5%)	0.042	20 (18.9%)	0.656
II	188	163 (86.7%)		43 (22.9%)	
III	98	83 (84.7%)		19 (19.4%)	
IV	0	0 (0.0%)		0 (0.0%)	
*Estrogen receptor*					
Negative	148	139 (93.9%)	<0.001	59 (39.9%)	<0.001
Positive	244	187 (76.6%)		23 (9.4%)	
*Progesterone receptor*					
Negative	192	177 (92.2%)	<0.001	63 (32.8%)	<0.001
Positive	200	149 (74.5%)		19 (9.5%)	
*HER2 expression*					
Negative (0–1^+^)	184	147 (79.9%)	0.021	34 (18.5%)	0.001
Equivocal (2^+^)	107	86 (80.4%)		14 (13.1%)	
Positive (3^+^)	101	93 (92.1%)		34 (33.7%)	
*Molecular classification*					
Luminal A	147	104 (70.7%)	<0.001	12 (8.2%)	<0.001
Luminal B	101	87 (86.1%)		11 (10.9%)	
HER2-enriched	71	66 (93.0%)		33 (46.5%)	
TNBC	73	69 (94.5%)		26 (35.6%)	

Abbreviations: HER2 = human epidermal growth factor receptor 2; TNBC = triple-negative breast cancer.

**Table 3:** Relationships between positive resistin expression and EGFR expression in 392 Chinese Han patients with breast cancer.

Patients	No.	EGFR expression			
		Negative *n* (%)	Positive *n* (%)	Not strongly positive *n* (%)	Strongly positive *n* (%)
Resistin negative	66	50 (75.8%)	16 (24.2%)	56 (84.8%)	10 (15.2%)
Resistin positive	326	138 (42.3%)	188 (57.7%)^∗^	209 (64.1%)	117 (35.9%)^∗∗^

^∗^ *P* < 0.001; ^∗∗^*P* = 0.001.

**Table 4:** Relationships between strongly positive resistin expression and EGFR expression in 392 Chinese Han patients with breast cancer.

Patients	No.	EGFR expression			
		Negative *n* (%)	Positive *n* (%)	Not strongly positive *n* (%)	Strongly positive *n* (%)
Not strongly positive for resistin	310	174 (56.1%)	136 (43.9%)	225 (72.6%)	85 (27.4%)
Strongly positive for resistin	82	14 (17.1%)	68 (82.9%)^∗^	40 (48.8%)	42 (51.2%)^∗∗^

^∗^ *P* < 0.001; ^∗∗^*P* < 0.001.

## Data Availability

All data generated or analyzed during this study are included in this published article.

## Abbreviations

EGFR: Epidermal growth factor receptor
IHC: Immunohistochemistry
MAPK: Mitogen-activated protein kinase
ERK1/2: Extracellular signal-regulated kinase 1/2
RFS: Relapse-free survival
OS: Overall survival.

## Supplementary Materials

Supplementary Materials Relationships between resistin expression and ERK1/2 expression in patients with breast cancer
