# Supplementary material for: Combined High Resistin and EGFR Expression Predicts a Poor Prognosis in Breast Cancer
Source: Biomed Res Int. 2020 Nov 28;2020:8835398. doi: 10.1155/2020/8835398 (PMC7719526; doi:10.1155/2020/8835398)
Supplement: Supplementary Materials — Relationships between resistin expression and ERK1/2 expression in patients with breast cancer [file 8835398.f1.pdf]

Combined high resistin and EGFR expression predicts a poor prognosis in breast cancer

Yue Zeng[1-2#], Chih-Hsin Tang[3–5#], Yan Wang[6], Hua-Jun Lu[7], Bi-Fei Huang[2], Qian Wang[2], Jun-Kang Shao[2], Lu-Lu Jin[8], Chao-Qun Wang[2*], Li-Song Teng[1*]

[1]Department of Surgical Oncology, the First Affiliated Hospital, Medical School of Zhejiang University, Hangzhou, Zhejiang, China

[2]Department of Pathology, Affiliated Dongyang Hospital of Wenzhou Medical University, Dongyang, Zhejiang, China

[3]Graduate Institute of Basic Medical Science, China Medical University, Taichung, Taiwan

[4]Department of Pharmacology, School of Medicine, China Medical University, Taichung, Taiwan

[5]Department of Biotechnology, College of Health Science, Asia University, Taichung, Taiwan

[6]Department of Medical Oncology, Affiliated Dongyang Hospital of Wenzhou Medical University, Dongyang, Zhejiang, China

[7]Department of Oncological Radiotherapy, Affiliated Dongyang Hospital of Wenzhou Medical University, Dongyang, Zhejiang, People's Republic of China

[8]Laboratory of Biomedicine, Affiliated Dongyang Hospital of Wenzhou Medical University, Dongyang, Zhejiang, People's Republic of China

[#]These authors contributed equally to this manuscript

[*]Corresponding author:

Chao-Qun Wang; Department of Pathology, Affiliated Dongyang Hospital of Wenzhou Medical University, 60 Wu Ning Xi Road, Dongyang 322100, China.

E-mail: chaoqunw869@163.com

Li-Song Teng; Department of Surgical Oncology, the First Affiliated Hospital, Medical School of Zhejiang University, 79 Qingchun Road, Hangzhou 310003, China.
E-mail: Lsteng@zju.edu.cn

Supplementary materials

IHC analyses detected ERK1/2 expression in 121 breast cancer cases. A case was considered to be ERK1/2-positive if the percentage of positive invasive cancer cells (nuclear and cytoplasmic staining) was ≥1%[1]. The rate of positive ERK1/2 expression in breast cancer tissue specimens was 65.3% (79/121). We found a significantly higher level of ERK1/2 expression in specimens from resistin-positive (68.1%, 79/116) compared with those that were resistin-negative (0.0%, 0/5; P<0.01, Table S1). Spearman correlation analysis revealed a significantly positive correlation between positive levels of resistin expression and ERK1/2-positive expression in breast cancer tissue specimens (r=0.285, P=0.002). No such correlation was observed between strongly positive resistin expression and ERK1/2 expression (Table S2).

Table S1. Relationships between positive resistin expression and ERK1/2 expression in 121 Chinese Han patients with breast cancer

		ERK1/2 expression	
Group	No.	Negative, n (%)	Positive, n (%)
Resistin-negative	5	5 (100.0%)	0 (0.0%)
Resistin-positive	116	37 (31.9%)	79 (68.1%)*

* P<0.01.

Table S2. Relationships between strongly positive resistin expression and ERK1/2 expression in 121 Chinese Han patients with breast cancer

		ERK1/2 expression	
Group	No.	Negative, n (%)	Positive, n (%)
Not strongly positive for resistin	68	27 (39.7%)	41 (60.3%)
Strongly positive for resistin	53	15 (28.3%)	38 (71.7%)*

* P=0.191.

References

1. Nguyen TH, Nguyen VH, Nguyen TL, Qiuyin C, Phung TH. Evaluations of Biomarker Status Changes between Primary and Recurrent Tumor Tissue Samples in Breast Cancer Patients. *BioMed research international* 2019;**2019**: 7391237.
